# Early-Life Antibiotic Exposure Associated With Varicella Occurrence and Breakthrough Infections: Evidence From Nationwide Pre-Vaccination and Post-Vaccination Cohorts

**DOI:** 10.3389/fimmu.2022.848835

**Published:** 2022-03-31

**Authors:** Teng-Li Lin, Yi-Hsuan Fan, Yi-Ling Chang, Hsiu J. Ho, Li-Lin Liang, Yi-Ju Chen, Chun-Ying Wu

**Affiliations:** ^1^ Department of Dermatology, Dalin Tzu Chi Hospital, Buddhist Tzu Chi Medical Foundation, Chiayi, Taiwan; ^2^ Department of Dermatology, Taichung Veterans General Hospital, Taichung, Taiwan; ^3^ Department of Pediatrics, Chung Shan Medical University Hospital, Taichung, Taiwan; ^4^ Institute of Biomedical Informatics and Research Center for Epidemic Prevention, National Yang Ming Chiao Tung University, Taipei, Taiwan; ^5^ Institute of Public Health, School of Medicine, National Yang Ming Chiao Tung University, Taipei, Taiwan; ^6^ School of Life Sciences, National Chung-Hsing University, Taichung, Taiwan; ^7^ Faculty of Medicine and Institute of Clinical Medicine, National Yang Ming Chiao Tung University, Taipei, Taiwan; ^8^ Division of Translational Research, Taipei Veterans General Hospital, Taipei, Taiwan; ^9^ Department of Public Health, China Medical University, Taichung, Taiwan; ^10^ National Institute of Cancer Research and Institute of Population Health Science, National Health Research Institutes, Miaoli, Taiwan

**Keywords:** varicella, breakthrough infection, vaccine, antibiotic, microbiota, dysbiosis, pediatric population

## Abstract

**Background:**

Antibiotic-driven dysbiosis may impair immune function and reduce vaccine-induced antibody titers.

**Objectives:**

This study aims to investigate the impacts of early-life antibiotic exposure on subsequent varicella and breakthrough infections.

**Methods:**

This is a nationwide matched cohort study. From Taiwan’s National Health Insurance Research Database, we initially enrolled 187,921 children born from 1997 to 2010. Since 2003, the Taiwan government has implemented a one-dose universal varicella vaccination program for children aged 1 year. We identified 82,716 children born during the period 1997 to 2003 (pre-vaccination era) and 48,254 children born from July 1, 2004, to 2009 (vaccination era). In the pre-vaccination era, 4,246 children exposed to antibiotics for at least 7 days within the first 2 years of life (Unvaccinated A-cohort) were compared with reference children not exposed to antibiotics (Unvaccinated R-cohort), with 1:1 matching for gender, propensity score, and non-antibiotic microbiota-altering medications. Using the same process, 9,531 children in the Vaccinated A-cohort and Vaccinated R-cohort were enrolled from the vaccination era and compared. The primary outcome was varicella. In each era, demographic characteristics were compared, and cumulative incidences of varicella were calculated. Cox proportional hazards model was used to examine associations.

**Results:**

In the pre-vaccination era, the 5-year cumulative incidence of varicella in the Unvaccinated A-cohort (23.45%, 95% CI 22.20% to 24.70%) was significantly higher than in the Unvaccinated R-cohort (16.72%, 95% CI 15.62% to 17.82%) (p<.001). In the vaccination era, a significantly higher 5-year cumulative incidence of varicella was observed in the Vaccinated A-cohort (1.63%, 95% 1.32% to 1.93%) than in the Vaccinated R-cohort (1.19%, 95% CI 0.90% to 0.45%) (p=0.006). On multivariate analyses, early-life antibiotic exposure was an independent risk factor for varicella occurrence in the pre-vaccination (adjusted hazard ratio [aHR] 1.92, 95% CI 1.74 to 2.12) and vaccination eras (aHR 1.66, 95% CI 1.24 to 2.23). The use of penicillins, cephalosporins, macrolides, or sulfonamides in infancy was all positively associated with childhood varicella regardless of vaccine administration.

**Conclusions:**

Antibiotic exposure in early life is associated with varicella occurrence and breakthrough infections.

## Introduction

The early-life microbiome has a fundamental role in human immunity. Indigenous microbiota provides crucial signals for maturation and modulation of the immune system (1, 2). In contrast, dysbiosis in infancy might cause stunting and dysregulation of immunity (3, 4). The composition of gut microbiota also correlates with vaccine immunogenicity (5). Evidence has suggested the association between early-life microbial colonization and sustainable vaccine-specific memory T-cells and antibody responses (6).

Exposure to medications, particularly antibiotics, is a common cause of dysbiosis (7, 8). Even short-term or low-dose antibiotics can disturb the delicate ecosystem of the infant microbiome (9, 10). Early-life antibiotic exposure has been linked to a higher risk of various conditions, including inflammatory bowel disease, type 2 diabetes, and atopic disorders (11–13). However, little is known about the effect of infantile antibiotic exposure on susceptibility to later-life infections. In addition, although antibiotic-driven dysbiosis has been found to impair vaccine responses (10, 14, 15), limitations are that most studies were conducted with a small sample size and in animal models or adults. Whether early-life antibiotic exposures decrease vaccine efficacy or increase the risk of breakthrough infections in the pediatric population remains to be elucidated.

Varicella was once associated with a significant impact on public health in Taiwan (16). Since the implementation of universal varicella vaccination (UVV) in 2003, disease transmission has been successfully controlled (17). However, varicella outbreaks among schoolchildren still occurred occasionally (18), and breakthrough infections continue to be reported despite high rates of national vaccination coverage (19). The present study was aimed to investigate the effect of early-life antibiotic exposure on childhood varicella risk and breakthrough infections.

## Materials and Methods

### Data Source

We conducted a nationwide cohort study using Taiwan’s National Health Insurance Research Database (NHIRD) from 1997 to 2013. The NHIRD contains detailed healthcare information from more than 99% of Taiwan’s population of 25 million people. Diagnoses are documented in the NHIRD using codes based on the International Classification of Diseases, Revision 9, Clinical Modification (ICD-9-CM). The accuracy of diagnosis in the NHIRD has been validated (20, 21), and the data have been used extensively in clinical epidemiology and health service research (22, 23). Personal information, including body weight, height, lifestyle, occupation, and cluster history, is unavailable from the database. This study has been approved by the ethical review board of Taichung Veterans General Hospital (No. CE20224B).

### Vaccination

The live attenuated varicella vaccine was approved for use in Taiwan in 1997. Two brands of OKA-strain varicella vaccines, Varivax (Merck) and Varilrix (GlaxoSmithKline) are available in Taiwan. The vaccines have been first provided free to children aged 1 year in Taipei City and Taichung City since 1998 and 1999, respectively. In 2003, the Taiwan government implemented the UVV program, targeting 1-year-old children. The self-paid second-dose booster has been recommended for children aged 4 to 6 years. Despite unavailable 2-dose vaccination rates, the cumulative coverage rate of at least one dose of varicella vaccine among children born after July 1, 2004, has reached more than 94% to date (24).

### Study Design and Population

From the NHIRD, children born from 1997 to 2010 were eligible for enrollment. Children born from 1997 to 2003 and living in regions other than Taipei City and Taichung City were considered unvaccinated (pre-vaccination era). Children born during July 1, 2004, and 2009 were deemed vaccinated (vaccination era). We excluded children with a follow-up period of less than one year, death registration, malignancy, immunodeficiency disorders, white blood cell disorders, transplantation, chemotherapy, or immunotherapy before varicella development. The diagnostic codes for these comorbidities are presented in **Supplemental Table 1**.

Early life, especially from conception to 2 years of age, is a critical window for microbiota development and immune maturation (25, 26). In the vaccination era, children who received antibiotics for at least 7 days within the first 2 years of life were included in the antibiotic cohort (Vaccinated A-cohort). The reference cohort (Vaccinated R-cohort) comprised children who had not received antibiotics. We identified the Unvaccinated A-cohort and the Unvaccinated R-cohort in the pre-vaccination era using the same process.

The index date was defined as the first day of the third year of life. All sampled children were followed up from age 2 years to the development of outcome of interest or death. Each child was followed up for a maximum of 5 years.

In each era, 1:1 matching of children in both cohorts was carried out for gender, propensity score, and non-antibiotic microbiota-altering medications. The propensity score was calculated *via* logistic regression model (27) that included infectious diseases, non-bacterial gastroenteritis, and constipation (**Supplemental Table 1**). These have been common pediatric comorbidities that promote intestinal dysbiosis. Histamine type-2 receptor antagonists (H2RAs), proton pump inhibitors (PPIs), and laxatives have been found to cause perturbation of gut microbiota (28). Non-antibiotic microbiota-altering medication exposure was defined as using any of these drugs for at least 7 days within the first 2 years of life.

### Outcome Measurement

The primary outcome was varicella with diagnostic code (ICD-9-CM code 052) in the NHIRD. Children with varicella before the index date were still censored during the follow-up. However, to evaluate the association between early-life antibiotic exposure and subsequent varicella, only varicella that occurred in children after 2 years of age was identified.

Breakthrough varicella has been defined as varicella occurring over 6 weeks after at least one dose of vaccination (17, 19). Since the age at varicella vaccination in Taiwan was previously reported to be 1 to 1.97 years (17), the identified varicella cases in the vaccination era were considered breakthrough events.

### Covariate Assessment

Demographic factors such as gender, comorbidities, and medication were considered potential confounders. Comorbidities were defined as diseases based on diagnostic codes (**Supplemental Table 1**) after the index date. Exposure to drugs related to dysbiosis, including H2RAs, PPIs, or laxatives, was defined as the use of such medications for at least 7 days within the first 2 years of life. Exposure to immunomodulatory drugs, such as systemic corticosteroids and disease-modifying antirheumatic drugs, was defined as using these drugs for more than 30 days per year on average. The aforementioned medication is listed in **Supplemental Table 2**.

### Statistical Analysis

We first analyzed the demographic data, comorbidities, and medications. The categorical variables and prevalence rates of varicella in the study cohorts of each era were compared using the chi-square test. The cumulative incidences of varicella were calculated using the Kaplan-Meier method. The differences in the full time-to-event distributions between the two cohorts of each era were tested *via* the 2-tailed log-rank test.

We next performed multivariate analyses with modified Cox proportional hazard models to determine whether antibiotic exposure is an independent risk factor for subsequent varicella. The adjusted variables were gender, hospital visit number during the follow-up period, and well-known factors for dysbiosis, including antibiotic exposure, use of non-antibiotic microbiota-altering medications, infectious diseases, non-bacterial gastroenteritis, and constipation. We also conducted sub-analyses to examine the risk of exposure to different antibiotics in early life on varicella development.

All data were managed *via* SAS 9.4 software (SAS Institute Inc., Cary, NC, USA) and the “cmprsk” package of R. The results are expressed as an estimated number with 95% confidence interval (CI).

## Results

### Demographic Characteristics of the Study Cohorts

**Figure 1:**
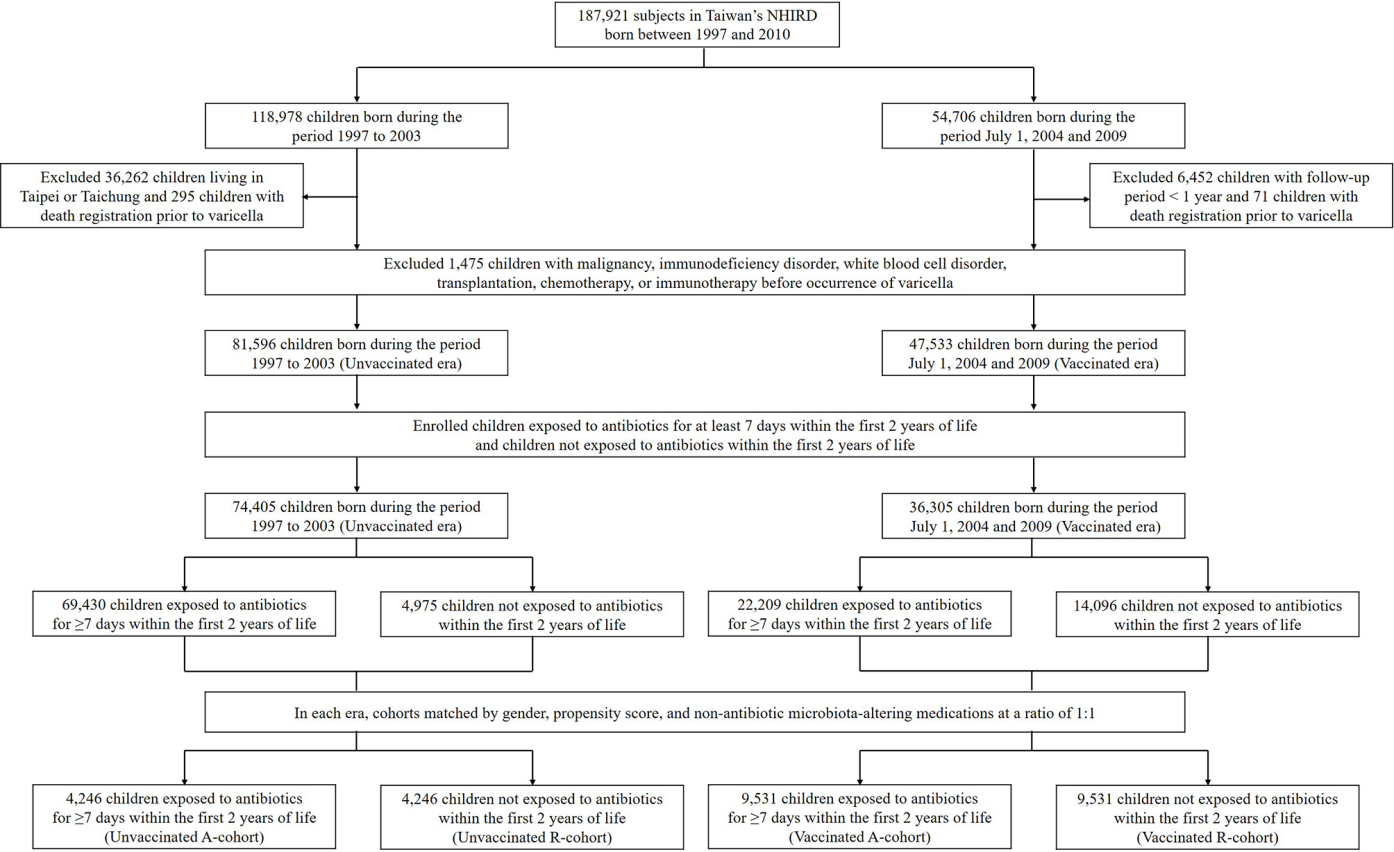
Flow chart of the patient selection process. NHIRD, National Health Insurance Research Database; A-cohort, antibiotic cohort; R-cohort, reference cohort.

We initially enrolled 187,921 children born from 1997 to 2010 from Taiwan’s NHIRD. Among them, 82,716 children not living in Taipei City or Taichung City were born from 1997 to 2003, and 48,254 children were born from July 1, 2004, to 2009. A total of 8,293 children with a follow-up period of less than 1 year or with comorbidities or therapy that may increase the risk of infections before the occurrence of varicella were excluded. Finally, 81,596 children were included in the pre-vaccination era group and 47,533 were included in the vaccination era group (**Figure 1**). The baseline characteristics of the children in both groups are presented in **Supplemental Table 3**.

In the pre-vaccination era, 69,430 children exposed to antibiotics for at least 7 days within the first 2 years were included in the Unvaccinated A-cohort, and 4,975 children in the reference group not exposed to antibiotics within the first 2 years of life were included in the Unvaccinated R-cohort. The baseline characteristics of children in both cohorts are shown in **Supplemental Table 4**. After matching for gender, propensity score, and non-antibiotic microbiota-altering medications at a ratio of 1:1, there were 4,246 children in each cohort (**Figure 1**). Using the same process, we selected subjects from the vaccination era, with 9,531 children each in the Vaccinated A-cohort and the Vaccinated R-cohort (**Figure 1**).

Demographic characteristics and comorbidities were comparable between the cohorts in each era, except higher numbers of hospital visits in both A-cohorts compared to the respective R-cohorts (median 72 vs. 59 in pre-vaccination era, and 77 vs.71 in vaccination era) (**Table 1**). In the Unvaccinated A-cohort, penicillins (59.6%) were most common, followed by cephalosporins (33.4%), macrolides (32.0%), and sulfonamides (22.7%). In the Vaccinated A-cohort, penicillins (61.1%) and cephalosporins (15.4%) were most common (**Supplemental Table 5**). Ages at varicella occurrence and hospitalization for varicella were comparable between the cohorts in each era.

**Table 1:** Demographic characteristics and outcomes of matched study subjects in antibiotic and reference cohorts in the pre-vaccination and vaccination eras.

	Pre-vaccination era			Vaccination era			
	Antibiotic cohort^a^(N=4,426)	Reference cohort^a^(N=4,426)	P-value	Antibiotic cohort^a^(N=9,531)	Reference cohort^a^ (N=9,531)	P-value	
Age, years							
Mean ± SD	3.0 ± 0.0	3.0 ± 0.0	NA	3.0 ± 0.0	3.0 ± 0.0	NA	
Median (IQR)	3.0 (3.0-3.0)	3.0 (3.0-3.0)	NA	3.0 (3.0-3.0)	3.0 (3.0-3.0)	NA	
Gender, N (%)			>.999			>.999	
Female	2,371 (53.6%)	2,371 (53.6%)		4,693 (49.2%)	4,693 (49.2%)		
Male	2,055 (46.4%)	2,055 (46.4%)		4,838 (50.8%)	4,838 (50.8%)		
Follow-up, years							
Mean ± SD	4.4 ± 1.3	4.5 ± 1.2	<.001	3.6 ± 1.3	3.5 ± 1.3	<.001	
Median (IQR)	5.0 (5.0-5.0)	5.0 (5.0-5.0)	<.001	4.0 (2.5-5.0)	3.7 (2.3-5.0)	<.001	
Hospital visits, N							
Mean ± SD	82.7 ± 59.2	70.5 ± 56.0	<.001	88.9 ± 58.3	81.0 ± 54.3	<.001	
Median (IQR)	72.0 (41.0-113.0)	59.0 (29.0-99.8)	<.001	77.0 (47.0-118.0)	71.0 (42.0-109.0)	<.001	
Antibiotic exposure, days							
Mean ± SD	39.2 ± 32.1	NA	NA	21.5 ± 24.5	NA	0.187	
Median (IQR)	29.0 (16.0-52.0)	NA	NA	15.0 (10.0-24.0)	NA	0.220	
Non-antibiotic microbiota-altering medication exposure,^b^ N (%)	270 (6.1%)	270 (6.1%)	>.999	1,000 (10.5%)	1,000 (10.5%)		>.999
Immunomodulatory drugs,^c^ N (%)							
Corticosteroids	13 (0.3%)	13 (0.3%)	>.999	29 (0.3%)	26 (0.3%)		0.787
DMARDs	1 (0.0%)	NA	>.999	1 (0.0%)	NA		>.999
Early-life infectious diseases,^d^ N (%)							
All infections^e^	2,162 (48.8%)	2,148 (48.5%)	0.782	7,777 (81.6%)	7,748 (81.3%)		0.602
Chronic sinusitis	51 (1.2%)	49 (1.1%)	0.920	72 (0.8%)	89 (0.9%)		0.205
Acute otitis media	82 (1.9%)	88 (2.0%)	0.699	559 (5.9%)	549 (5.8%)		0.781
Acute upper respiratory infections	3,846 (86.9%)	3,847 (86.9%)	>.999	9,520 (99.9%)	9,523 (99.9%)		0.646
Acute bronchitis/bronchiolitis	2,460 (55.6%)	2,447 (55.3%)	0.797	8,502 (89.2%)	8,474 (88.9%)		0.531
Pneumonia	411 (9.3%)	415 (9.4%)	0.913	2,563 (26.9%)	2,569 (27.0%)		0.935
Urinary tract infections	29 (0.7%)	32 (0.7%)	0.797	142 (1.5%)	140 (1.5%)		0.952
Meningitis	6 (0.1%)	7 (0.2%)	>.999	4 (0.0%)	8 (0.1%)		0.386
Sepsis	49 (1.1%)	48 (1.1%)	>.999	60 (0.6%)	67 (0.7%)		0.593
Cellulitis/abscess	162 (3.7%)	163 (3.7%)	>.999	927 (9.7%)	955 (10.0%)		0.512
Impetigo	88 (2.0%)	95 (2.1%)	0.654	221 (2.3%)	220 (2.3%)		>.999
Comorbidities,^f^ N (%)							
Congenital anomalies of heart	76 (1.7%)	53 (1.2%)	0.051	187 (2.0%)	117 (1.2%)		<.001
Kawasaki disease	9 (0.2%)	12 (0.3%)	0.662	42 (0.4%)	15 (0.2%)		<.001
Non-bacterial gastroenteritis	2,414 (54.5%)	2,419 (54.7%)	0.932	6,303 (66.1%)	6,250 (65.6%)		0.427
Constipation	851 (19.2%)	855 (19.3%)	0.936	2,189 (23.0%)	2,176 (22.8%)		0.836
Intussusception	190 (4.3%)	173 (3.9%)	0.391	317 (3.3%)	297 (3.1%)		0.436
Appendicitis	20 (0.5%)	22 (0.5%)	0.877	33 (0.3%)	25 (0.3%)		0.357
Febrile convulsion	10 (0.2%)	19 (0.4%)	0.137	86 (0.9%)	98 (1.0%)		0.415
Epilepsy	37 (0.8%)	34 (0.8%)	0.812	75 (0.8%)	51 (0.5%)		0.040
Outcomes							
Varicella, N (%)	1,038 (23.5%)	740 (16.7%)	<.001	117 (1.2%)	75 (0.8%)		0.003
Age at varicella onset, years							
Mean ± SD	5.3 ± 1.2	5.2 ± 1.2	0.664	5.1 ± 1.3	5.3 ± 1.3		0.187
Median (IQR)	5.2 (4.4~6.0)	5.2 (4.3~6.1)	0.688	5.0 (3.8~6.0)	5.3 (4.4~6.2)		0.220
Hospitalization for varicella	7 (0.2%)	3 (0.1%)	0.343	1 (0.0%)	2 (0.0%)		>.999

N, number; SD, standard deviation; IQR, interquartile range; NA, not available; DMARDs, disease-modifying antirheumatic drugs.

a In each era, the antibiotic cohort and the reference cohort were matched by gender, propensity score, and non-antibiotics microbiota-altering medications at a ratio of 1:1.

b Non-antibiotic microbiota-altering medication exposure refer to the use of histamine type-2 receptor antagonists, proton pump inhibitors, or laxatives for at least 7 days within the first 2 years of life.

c Immunomodulatory drug exposure refers to the use of corticosteroids or DMARDs for at least 30 days after the index date.

d Early-life infectious diseases refer to infections with diagnostic code recorded in the database at least once before the index date.

e All infections include infectious diseases with codes 001-039 but 042 in the International Classification of Diseases, Ninth Revision [ICD-9].

f Comorbidities refer to comorbidities with diagnostic code recorded in the database at least once after the index date.

### Cumulative Incidences of Varicella

A significantly higher 5-year cumulative incidence of varicella was observed in the pre-vaccination era group (22.22%, 95% confidence interval [CI] 21.94-22.51%) than in the vaccination era group (1.40%, 95% CI 1.27-1.53%) (p<.001) (**Supplemental Figure 1**).

**Figure 2:**
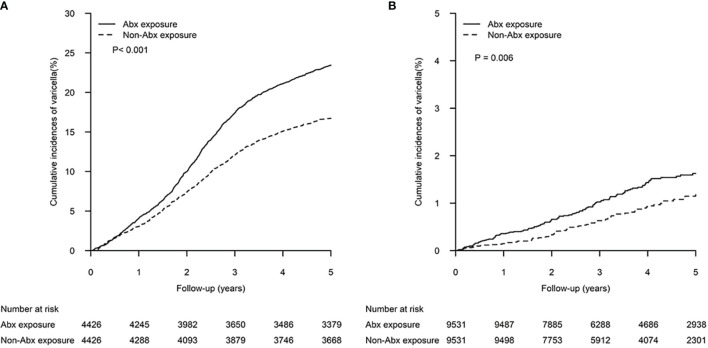
Cumulative incidences of varicella in patients exposed to antibiotics within the first 2 years of life and matched controls. The differences between the two study cohorts in the **(A)** pre-vaccination era and **(B)** vaccination era were determined by log-rank test.

In the pre-vaccination era, the 5-year cumulative incidence of varicella in the Unvaccinated A-cohort (23.45%, 95% CI 22.20-24.70%) was significantly higher than in the Unvaccinated R-cohort (16.72%, 95% CI 15.62-17.82%) (p<.001) (**Figure 2A**). In the vaccination era, a significantly higher 5-year cumulative incidence of varicella was observed in the Vaccinated A-cohort (1.63%, 95% 1.32-1.93%) than in the Vaccinated R-cohort (1.19%, 95% CI 0.90-1.45%) (p=0.006) (**Figure 2B**).

### Multivariate Analyses

In the pre-vaccination era, antibiotic exposure for at least 7 days within the first 2 years of life was independently associated with varicella occurrence (adjusted hazard ratio [aHR] 1.92, 95% CI 1.74-2.12). This risk was weaker but still significant among children born in the vaccination era (aHR 1.66, 95% CI 1.24-2.23) (**Table 2**).

**Table 2:** Multivariate analyses of antibiotic effects for varicella in the pre- vaccination and vaccination eras.

	aHR (95% CI)^a^	
	Pre-vaccination era	Vaccination era
Antibiotic exposures	1.92 (1.74-2.12)	1.66 (1.24-2.23)
Penicillins^b^	1.47 (1.31-1.66)	1.28 (0.94-1.74)
Cephalosporins^b^	1.19 (1.04-1.36)	1.41 (0.88-2.26)
Macrolides^b^	1.46 (1.28-1.67)	1.25 (0.67-2.34)
Sulfonamides^b^	1.27 (1.09-1.48)	1.27 (0.59-2.73)

aHR, adjusted hazard ratio; CI, confidence interval.

a Adjusted for gender, hospital visit number, antibiotic exposure, non-antibiotic microbiota-altering medication, infections within the first 2 years of life, non-bacterial gastroenteritis, and constipation.

b Substitution of specific type of antibiotic for any type of antibiotic in the same model on multivariate analyses.

Further analyses demonstrated that exposure to a specific type of the commonly-used antibiotics, including penicillins (aHR 1.47, 95% CI 1.31-1.66), cephalosporins (aHR 1.19, 95% CI 1.04-1.36), macrolides (aHR 1.46, 95% CI 1.28-1.67), and sulfonamides (aHR 1.27, 95% CI 1.09-1.48), were also independent risk factors for varicella occurrence in the pre-vaccination era. However, exposure to these antibiotics in the vaccination era was positively associated with subsequent varicella but without statistical significance (**Table 2**).

## Discussion

This nationwide cohort study suggests that antibiotic exposure early in life is an independent risk factor for childhood varicella. Even though herd immunity has been reached in the vaccination era, a significantly higher incidence of breakthrough varicella is observed in children exposed to antibiotics in early life. The present study adds to the mounting evidence that antibiotic-driven dysbiosis during infancy may cause sequelae linked with immune dysfunction, including increased susceptibility to infections.

Commensal-pathogen interactions involve the direct microbiota-related colonization resistance and the indirect microbiome-mediated immune modulation (29). Commensal microbiota can limit colonization of the invading pathogen through upregulating epithelial barrier function, competition for specific resources, and bactericidal or bacteriostatic effects (29, 30). Eubiotic microbiota also supports healthy immune development, shaping optimal innate and acquired immune responses against infective challenges (1, 2).

Evidence has demonstrated that a decrease in bacterial taxa, vacant nutrient niches, and metabolic environment changes after antibiotic administration predispose individuals to certain infections (31, 32), whereas the commensals may progressively return to baseline following antibiotic cessation (33). On the other hand, antibiotic-driven dysbiosis, especially in early life, might result in enduring immune alterations and long-lasting health impacts (3, 4). Animal studies have demonstrated that infant mice exposed to antibiotics had reduced and dysfunctional interferon-γ-producing CD8 T cells, resulting in subsequent increased mortality from vaccinia virus infection (34). In humans, children exposed to early-life antibiotics have been found to exhibit lower infection-induced cytokines, including interleukin 1β, interferon α, interferon γ, tumor necrosis factor α, and IP10 protein (35). Our results align with these immunological findings and support the microbiome-immune-infection axis theory. Early-life antibiotic exposure is associated with dysbiosis and impaired anti-infectious immunity and increases susceptibility to future varicella infections.

The role of the microbiome in the modulation of vaccine immunogenicity has recently been addressed (5). Several observational studies have documented the correlation between microbiota composition, such as the abundance of Bifidobacterium and Bacteroides species, and vaccine responses (6, 36, 37). Immunomodulatory molecules derived from microbiota, such as flagellin, peptidoglycan, and lipopolysaccharides, regulate T cell priming and immunoglobulin production in response to antigenic stimulation (36, 38, 39). Increasing data also suggests that epitopes encoded by the microbiota can cross-reactive with pathogen-encoded epitopes, presumably with vaccine-encoded epitopes (40, 41).

Despite the association between microbiome and vaccine responses, controversy exists over the influence of microbial perturbation on immunization. Antibiotic-driven dysbiosis impairs vaccine immunogenicity in infant mice but not in adult mice (14). From human research, adults with low pre-existing immunity have been found to present markedly reduced post-vaccination antibody titers after experiencing antibiotic treatment (10). Nevertheless, antibiotic exposure in early life has not significantly affected immunogenicity induced by routine infant vaccines, while sample sizes of these studies were modest (42, 43). Additionally, effects of prebiotics or probiotics on vaccine response are variable, depending on the antigens, probiotic strains, and population (44–46). To date, none of these microbiota-targeted interventions have been transferred from research into clinical practice. Our study assessed incidence of breakthrough varicella among children exposed to early-life antibiotics. Although the UVV program has provided robust protection, infantile antibiotic exposure was still an independent risk factor for childhood breakthrough varicella. Such risk might result from increased varicella pathogenicity following antibiotic exposure overwhelming the vaccine protective efficacy or alteration of vaccine responses induced by antibiotic-driven dysbiosis. Further studies are needed to clarify the effects of early-life antibiotic exposure on immunization and vaccine efficacy.

The microbiota changes related to antibiotics depend on the type of antibiotic used. Previous studies have suggested that almost all types of antibiotics affect gut microbiota. The penicillin family of antibiotics, such as amoxicillin, piperacillin, and ticarcillin, may increase the abundance of Enterococcus spp. and decrease the abundance of anaerobes (47). Cephalosporins, quinolones, and sulfonamides have been associated with abundant Enterobacteriaceae except for Escherichia coli (47). Macrolide treatment has been linked to long-term gut microbiota perturbations among pre-school children, including depletion of Actinobacteria and increases in Bacteroidetes and Proteobacteria (48). The antimicrobial spectrum also influences the impact of antibiotics on the immune response to vaccination. An adult study has demonstrated that the proportion of vaccinees with a more than 2-fold anti-rotavirus antibody titer by 7 days post-vaccination was significantly higher among subjects treated with vancomycin only than those treated with broad-spectrum antibiotics (15). In the present study, early-life exposure to penicillins, cephalosporins, macrolides, or sulfonamides were all independent risk factors for childhood varicella in the pre-vaccination era. The risk of breakthrough varicella due to exposure to these antibiotics in the vaccination era was also observed, although without statistical significance owing to the small number of cases. Relationship between the risk and antimicrobial spectrum of the administered antibiotic remains to be elucidated, since we only examined the effects of using different types of antibiotics rather than the specific antibiotic on varicella occurrence. Overall, caution is warranted in prescribing any type of antibiotic to infants despite their benefits. It should also be taken into account the effects on the human microbiome when administering antibiotic therapy.

Our study has several strengths. The population-based cohort study design enabled us to assess the association between antibiotic exposure and varicella infections. By utilizing the nationwide NHIRD, we enrolled a large sample size, which prevented selection bias, allowing us to identify relatively rare conditions such as post-vaccination infection, and provide reliability in terms of statistics with a smaller margin of error.

Despite these strengths, there are several limitations. First, as this was an observational study, we could only report an association between antibiotic exposure and subsequent varicella but could not infer causality. Second, patient-specific information such as lifestyle, contact history, seeking healthcare in private practice, and over-the-counter medication use was unavailable from the NHIRD. To minimize biases, cohorts possessed comparable characteristics after matching gender, propensity score, and non-antibiotic microbiota-altering medications. We also performed multivariable analyses to adjust for potential confounders. Third, the specific date of vaccination, the total number of varicella vaccines administered, whether concomitant vaccinations were used or not, and the interval from antibiotic exposure to vaccination were not recorded in the dataset. Therefore, it is difficult to assess the effects of antibiotic exposure on immunization. Instead, we reported the association between antibiotic therapy in infancy and varicella during childhood regardless of herd immunity. Finally, as our study focused on varicella, the generalizability of our results may be limited. Nevertheless, it provided valuable information on the microbiome-immune-infection axis theory.

## Conclusions

In conclusion, children exposed to antibiotics in infancy are associated with varicella later in life. Antibiotic exposure is an independent risk factor for varicella occurrence, even though herd immunity has been reached. These findings suggest caution when administering antibiotics in early life to prevent increased infection susceptibility and poor vaccine efficacy.

## Data Availability Statement

The data analyzed in this study is subject to the following licenses/restrictions: All researchers who wish to use the NHIRD and its data subsets are required to sign a written agreement declaring that they have no intention of attempting to obtain information that could potentially violate the privacy of patients or care providers. Requests to access these datasets should be directed to Center for Biomedical Resources of NHRI, https://nhird.nhri.org.tw/en/Data_Protection.html.

## Ethics Statement

The studies involving human participants were reviewed and approved by the ethical review board of Taichung Veterans General Hospital (No. CE20224B). Written informed consent from the participants’ legal guardian/next of kin was not required to participate in this study in accordance with the national legislation and the institutional requirements.

## Author Contributions

Study concept and design: T-LL, Y-HF, Y-JC, C-YW. Statistical analysis: Y-LC, HH. Analysis and interpretation of data: T-LL, L-LL, Y-JC. Drafting of the manuscript: T-LL, Y-JC. Critical revision of the manuscript for important intellectual content: T-LL, C-YW, Y-JC. All authors read and approved the final manuscript.

## Funding

This work is supported in part by the Ministry of Science and Technology, Taiwan (MOST 109-2327-B-010-005; 109-2410-H-110-016-MY2).

## Conflict of Interest

The authors declare that the research was conducted in the absence of any commercial or financial relationships that could be construed as a potential conflict of interest.

## Publisher’s Note

All claims expressed in this article are solely those of the authors and do not necessarily represent those of their affiliated organizations, or those of the publisher, the editors and the reviewers. Any product that may be evaluated in this article, or claim that may be made by its manufacturer, is not guaranteed or endorsed by the publisher.
