# Supplementary material for: Early-Life Antibiotic Exposure Associated With Varicella Occurrence and Breakthrough Infections: Evidence From Nationwide Pre-Vaccination and Post-Vaccination Cohorts
Source: Front Immunol. 2022 Mar 31;13:848835. doi: 10.3389/fimmu.2022.848835 (PMC9011717; doi:10.3389/fimmu.2022.848835)
Supplement: Supplementary file 1 [file DataSheet_1.pdf]

Supplementary Material

Title: Early-life antibiotic exposure associated with varicella occurrence and breakthrough infections: evidence from nationwide pre-vaccination and post-vaccination cohorts

Authors:
Teng-Li Lin, MD, Yi-Hsuan Fan, MD, Yi-Ling Chang, MS, Hsiu J. Ho, PhD, Li-Lin Liang, PhD, Yi-Ju Chen, MD, PhD, Chun-Ying Wu, MD, MPH, PhD

Supplemental Figure 1. Cumulative incidences of varicella in patients in the pre-vaccination era and the vaccination era.
Supplemental Table 1. Codes for varicella and comorbidities in our analysis
Supplemental Table 2. The commonly used systemic antibiotics, H2RAs, PPIs, laxatives, corticosteroids and DMARDs in Taiwan
Supplemental Table 3. Demographic characteristics and outcomes of study subjects in the pre-vaccination and vaccination eras
Supplemental Table 4. Baseline demographic characteristics and outcomes of the study subjects in the antibiotic and reference cohorts in the pre-vaccination and vaccination eras
Supplemental Table 5. Antibiotic exposure in the matched study subjects among antibiotic cohorts in the pre-vaccination era and the vaccination eras

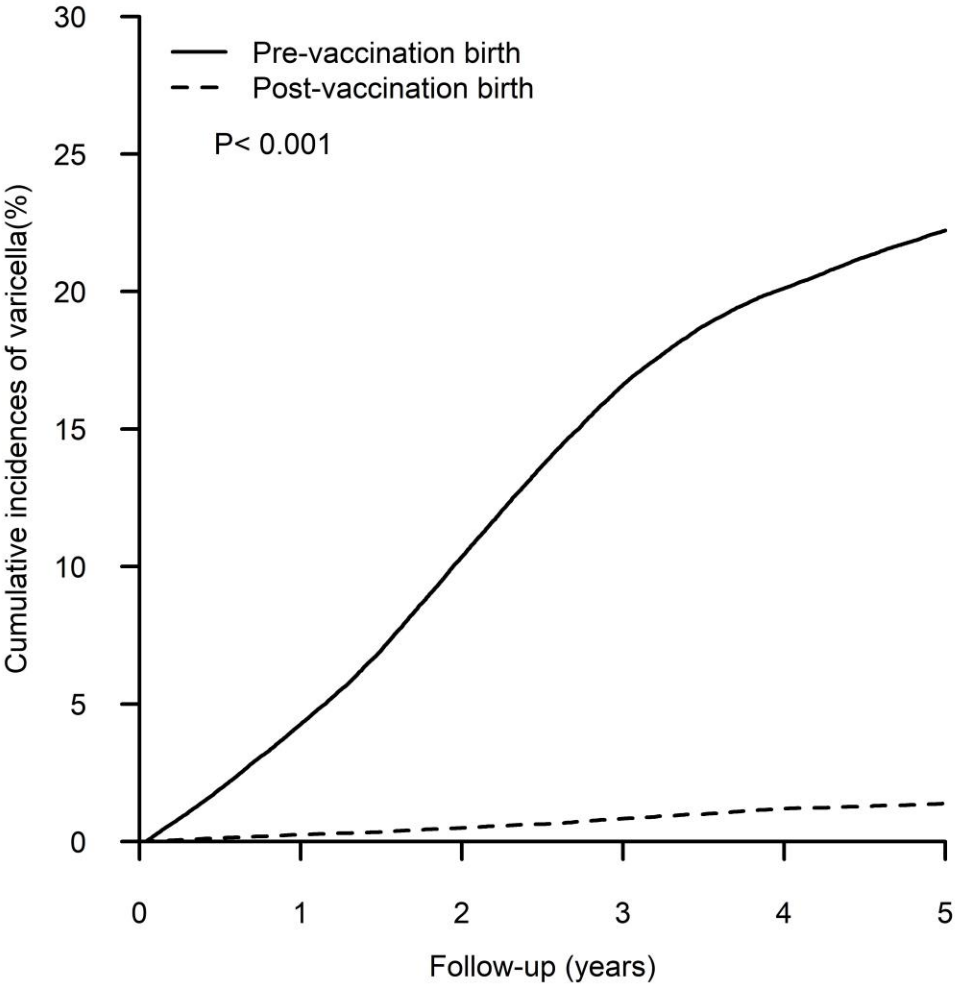

Number at risk

Pre-vaccination birth	81596	78084	73094	67956	65069	63321
Post-vaccination birth	47533	47323	39092	30676	22159	13378

Supplemental Figure 1. Cumulative incidences of varicella in patients in the pre-vaccination era and the vaccination era. The differences between patients in these two era were determined by log-rank test.

Supplemental Table 1. Codes for varicella and comorbidities in our analysis

	ICD-9-CM code
Varicella	052
Comorbidities other than infections	
Malignancy	140-208
Immunodeficiency disorders	042, 279.0-279.3
White blood cell disorders	284, 288
Transplantation	V42
Chemotherapy or immunotherapy	V58.1
Congenital anomalies of heart	745, 746
Kawasaki disease	446.1
Non-bacterial gastroenteritis	558.9
Constipation	564.00, 564.09
Intussusception	560
Appendicitis	540-5432
Febrile convulsion	780.31, 780.31
Epilepsy and recurrent seizures	345
Tourette syndrome/Tics	307.2
Infections	
All infections	001-139 (not include 042)
Chronic sinusitis	473
Acute otitis media	381
Acute upper respiratory infections	460-466
Acute bronchitis/bronchiolitis	490, 491
Pneumonia	480-486
Urinary tract infection	590, 595, 597
Meningitis	320.7, 320.9, 321.1, 321.2, 322
Sepsis	771.81, 995.91, 995.92
Cellulitis/Abscess	681, 682
Impetigo	684

Abbreviations: ICD-9-CM, International Classification of Diseases, Revision 9, Clinical Modification.

Supplemental Table 2. The commonly used systemic antibiotics, H2RAs, PPIs, laxatives, corticosteroids and DMARDs in Taiwan

Medication type	Drug
Antibiotics	Penicillin; Cephalosporin; Tetracycline; Macrolides; Aminoglycosides; Polypeptides; Quinolones; Sulfonamides; Anti-urinary infectives; Antifungals Antitrichomonal agents and anticandidal agents; Antitubercular agents; Combined regimens
H2RAs	Cimetidine, Famotidine, Nizatidine, Ranitidine, Roxatidine
PPIs	Omeprazole; Lansoprazole; Esomeprazole; Pantoprazole; Dexlansoprazole; Rabeprazole
Laxatives	Sterculia and Frangula; Psyllium husk; Methylcellulose; Polycarbophil; Magnesium oxide; Magnesium hydroxide; Polyethylene glycol-electrolyte solution; Monobasic sodium phosphate; Lactulose; Sorbitol; Sodium phosphate; Bisacodyl; Sennoside A+B; Castor Oil; Mineral oil; Glycerin; Dioctyl sodium sulfosuccinate
Corticosteroids	Betamethasone; Cortisone Acetate; Dexamethasone; Fludrocortisone Acetate; Hydrocortisone; Methylprednisolone; Prednisolone
DMARDs	Methotrexate; Cyclosporine; Azathioprine; Acitretin; Leflunomide; Hydroxychloroquine; Mycophenolate; Auranofin; Cyclophosphamide; Gold sodium thiomalate; D-penicillamine; Sulfasalazine

Abbreviations: H2RAs, histamine type-2 receptor antagonists; PPIs, proton pump inhibitors; DMARDs, disease-modifying antirheumatic drugs.

Supplemental Table 3. Demographic characteristics and outcomes of study subjects in the pre-vaccination and vaccination eras

	Pre-vaccination era (N=81,596)	Vaccination era (N=47,533)	P-value
Age, years			
Mean±SD	3.0±0.0	3.0±0.0	NA
Median (IQR)	3.0 (3.0-3.0)	3.0 (3.0-3.0)	NA
Gender, N (%)			0.910
Female	38,953 (47.7%)	22,708 (47.8%)	
Male	42,643 (52.3%)	24,825 (52.2%)	
Follow-up, years			
Mean±SD	4.4±1.3	3.6±1.3	<.001
Median (IQR)	5.0 (5.0-5.0)	3.8 (2.4-5.0)	<.001
Hospital visits, N			
Mean±SD	93.5±63.1	86.4±58.5	<.001
Median (IQR)	83.0 (48.0-125.0)	75.0 (44.0-116.0)	<.001
Medication exposure, N (%)			
Antibiotics[a]	69,430 (85.1%)	22,209 (46.7%)	<.001
H_2RAs[a]	1,651 (2.0%)	617 (1.3%)	<.001
PPIs[a]	10 (0.0%)	32 (0.1%)	<.001
Laxatives[a]	12,662 (15.5%)	5,511 (11.6%)	<.001
Corticosteroids[b]	313 (0.4%)	153 (0.3%)	0.083
DMARDs[b]	5 (0.0%)	5 (0.0%)	0.591
Early-life infectious diseases,[c] N (%)			
All infections[d]	58,023 (71.1%)	38,832 (81.7%)	<.001
Acute sinusitis	43,115 (52.8%)	33,387 (70.2%)	<.001
Chronic sinusitis	3,343 (4.1%)	890 (1.9%)	<.001
Acute otitis media	6,070 (7.4%)	4,067 (8.6%)	<.001
Acute upper respiratory infections	79,540 (97.5%)	46,968 (98.8%)	<.001
Acute bronchitis/bronchiolitis	64,708 (79.3%)	41,336 (87.0%)	<.001
Pneumonia	21,274 (26.1%)	16,804 (35.4%)	<.001
Urinary tract infection	1,694 (2.1%)	1,174 (2.5%)	<.001
Meningitis	556 (0.7%)	134 (0.3%)	<.001
Sepsis	2,276 (2.8%)	757 (1.6%)	<.001
Cellulitis/abscess	6,001 (7.4%)	5,746 (12.1%)	<.001
Impetigo	3,288 (4.0%)	1,432 (3.0%)	<.001
Comorbidities,[e] N (%)			

Congenital anomalies of heart	1,511 (1.9%)	790 (1.7%)	0.014
Kawasaki disease	224 (0.3%)	167 (0.4%)	0.018
Non-bacterial gastroenteritis	49,905 (61.2%)	31,276 (65.8%)	<.001
Constipation	18,492 (22.7%)	11,362 (23.9%)	<.001
Intussusception	4,185 (5.1%)	1,674 (3.5%)	<.001
Appendicitis	470 (0.6%)	129 (0.3%)	<.001
Febrile convulsion	295 (0.4%)	450 (0.9%)	<.001
Epilepsy	804 (1.0%)	314 (0.7%)	<.001
Tourette syndrome	378 (0.5%)	353 (0.7%)	<.001
Allergic diseases, N (%)			
Varicella, N (%)	18,133 (22.2%)	482 (1.0%)	<.001
Age at varicella onset, years	73 (25.1%)	236 (20.3%)	0.086
Mean±SD	5.2±1.2	5.2±1.3	0.582
Median (IQR)	5.1 (4.3-6.0)	5.1 (4.0-6.2)	0.650
Hospitalization for varicella	105 (0.1%)	13 (0.0%)	<.001

Abbreviations: N, number; SD, standard deviation; IQR, interquartile range; NA, not available; H_2RAs, histamine type-2 receptor antagonists; PPIs, proton pump inhibitors; DMARDs, disease-modifying antirheumatic drugs.

[a]Exposure to antibiotics, H_2RAs, PPIs, and laxatives refers to the use of any of these drugs for at least 7 days within the first 2 years of life..

[b]Exposure to immunomodulatory drugs refers to the use of corticosteroids or DMARDs for at least 30 days after the index date.

[c]Early-life infectious diseases refer to infections with diagnostic code recorded in the database at least once before the index date.

[d]All infections include infectious diseases with codes 001-039 but 042 in the International Classification of Diseases, Ninth Revision [ICD-9]

[e]Comorbidities refer to comorbidities with diagnostic code recorded in the database at least once after the index date.

Supplemental Table 4. Baseline demographic characteristics and outcomes of the study subjects in the antibiotic and reference cohorts in the pre-vaccination and vaccination eras

	Pre-vaccination era			Vaccination era		
	Antibiotic cohort (N=69,430)	Reference cohort (N=4,975)	P-value	Antibiotic cohort (N=22,209)	Reference cohort (N=14,096)	P-value
Age, years						
Mean±SD	3.0±0.0	3.0±0.0	NA	3.0±0.0	3.0±0.0	NA
Median (IQR)	3.0 (3.0-3.0)	3.0 (3.0-3.0)	NA	3.0 (3.0-3.0)	3.0 (3.0-3.0)	NA
Gender, N (%)			<.001			<.001
Female	32,487 (46.8%)	2,655 (53.4%)		9,974 (44.9%)	7,231 (51.3%)	
Male	36,943 (53.2%)	2,320 (46.6%)		12,235 (55.1%)	6,865 (48.7%)	
Follow-up, years						
Mean±SD	4.4±1.3	4.6±1.1	<.001	3.6±1.3	3.5±1.3	<.001
Median (IQR)	5.0 (5.0-5.0)	5.0 (5.0-5.0)	<.001	3.9 (2.4-5.0)	3.7 (2.4-5.0)	<.001
Hospital visits, N						
Mean±SD	95.6±63.0	65.1±56.0	<.001	94.3±60.4	74.1±53.2	<.001
Median (IQR)	85.0 (50.0-127.0)	53.0 (23.0-94.0)	<.001	83.0 (51.0-125.0)	64.0 (35.0-101.0)	<.001
Antibiotic exposure, days						
Mean±SD	46.7±37.8	NA	NA	25.3±26.9	NA	NA
Median (IQR)	36.0 (20.0-62.0)	NA	NA	17.0 (11.0~30.0)	NA	NA
Non-antibiotic microbiota-altering medication exposure,[a] N (%)	13,299 (19.2%)	270 (5.4%)	<.001	3,773 (17.0%)	1,131 (8.0%)	<.001
Histamine type-2 receptor antagonists	1,585 (2.3%)	16 (0.3%)	<.001	445 (2.0%)	84 (0.6%)	<.001
Propton pump inhibitors	9 (0.0%)	NA	0.892	27 (0.1%)	5 (0.0%)	0.012
Laxatives	11,768 (16.9%)	251 (5.0%)	<.001	3,365 (15.2%)	1,036 (7.3%)	<.001
Immunomodulatory drugs,[b] N (%)						

Corticosteroids	278 (0.4%)	13 (0.3%)	0.161	106 (0.5%)	27 (0.2%)	<.001
DMARDs	5 (0.0%)	NA	>.999	4 (0.0%)	NA	0.280
Early-life infectious diseases,[c] N (%)						
All infections[d]	51,342 (73.9%)	2,148 (43.2%)	<.001	19,955 (89.9%)	9,662 (68.5%)	<.001
Chronic sinusitis	3,197 (4.6%)	49 (1.0%)	<.001	692 (3.1%)	92 (0.7%)	<.001
Acute otitis media	5,702 (8.2%)	88 (1.8%)	<.001	2,757 (12.4%)	564 (4.0%)	<.001
Acute upper respiratory infections	68,713 (99.0%)	3,847 (77.3%)	<.001	22,198 (100.0%)	13,558 (96.2%)	<.001
Acute bronchitis/bronchiolitis	57,240 (82.4%)	2,447 (49.2%)	<.001	20,872 (94.0%)	10,634 (75.4%)	<.001
Pneumonia	19,847 (28.6%)	415 (8.3%)	<.001	10,905 (49.1%)	2,680 (19.0%)	<.001
Urinary tract infections	1,572 (2.3%)	32 (0.6%)	<.001	796 (3.6%)	148 (1.0%)	<.001
Meningitis	528 (0.8%)	7 (0.1%)	<.001	108 (0.5%)	8 (0.1%)	<.001
Sepsis	2,115 (3.0%)	48 (1.0%)	<.001	579 (2.6%)	67 (0.5%)	<.001
Cellulitis/abscess	5,396 (7.8%)	163 (3.3%)	<.001	3,478 (15.7%)	1,061 (7.5%)	<.001
Impetigo	2,971 (4.3%)	95 (1.9%)	<.001	895 (4.0%)	251 (1.8%)	<.001
Comorbidities,[e] N (%)						
Congenital anomalies of heart	1,369 (2.0%)	60 (1.2%)	<.001	476 (2.1%)	158 (1.1%)	<.001
Kawasaki disease	198 (0.3%)	12 (0.2%)	0.670	117 (0.5%)	20 (0.1%)	<.001
Non-bacterial gastroenteritis	43,189 (62.2%)	2,457 (49.4%)	<.001	15,276 (68.8%)	8,669 (61.5%)	<.001
Constipation	16,095 (23.2%)	886 (17.8%)	<.001	5,818 (26.2%)	2,917 (20.7%)	<.001
Intussusception	3,681 (5.3%)	177 (3.6%)	<.001	871 (3.9%)	420 (3.0%)	<.001
Appendicitis	417 (0.6%)	23 (0.5%)	0.257	72 (0.3%)	37 (0.3%)	0.343
Febrile convulsion	247 (0.4%)	19 (0.4%)	0.861	247 (1.1%)	118 (0.8%)	0.012
Epilepsy	37 (0.8%)	34 (0.8%)	0.812	187 (0.8%)	72 (0.5%)	<.001
Tourette syndrome	333 (0.5%)	19 (0.4%)	0.388	183 (0.8%)	92 (0.7%)	0.076
Outcomes						
Varicella	15,901 (22.9%)	780 (15.7%)	<.001	250 (1.1%)	120 (0.9%)	0.013

Age at varicella onset, years						
Mean±SD	5.2±1.2	5.3±1.2	0.071	5.1±1.3	5.4±1.3	0.054
Median (IQR)	5.1 (4.2-6.0)	5.2 (4.3-6.1)	0.050	5.1 (3.8-6.1)	5.3 (4.6-6.2)	0.065
Hospitalization for varicella	97 (0.1%)	3 (0.1%)	0.202	7 (0.0%)	3 (0.0%)	0.804

Abbreviations: N, number; SD, standard deviation; IQR, interquartile range; NA, not available; DMARDs, disease-modifying antirheumatic drugs.

[a]Non-antibiotic microbiota-altering medication exposure refers to the use of histamine type-2 receptor antagonists, proton pump inhibitors, or laxatives for at least 7 days within the first 2 years of life. For each type of medication in the following 3 rows, administration was for at least 7 days within the first 2 years of life.

[b]Immunomodulatory drug exposure refers to the use of corticosteroids or DMARDs for at least 30 days after the index date.

[c]Early-life infectious diseases refer to infections with diagnostic code recorded in the database at least once before the index date.

[d]All infections include infectious diseases with codes 001-039 but 042 in the International Classification of Diseases, Ninth Revision [ICD-9].

[e]Comorbidities refer to comorbidities with diagnostic code recorded in the database at least once after the index date.

Supplemental Table 5. Antibiotic exposure in the matched study subjects among antibiotic cohorts in the pre-vaccination era and the vaccination eras

	Unvaccinated Antibiotic cohort (N=69,430)	Vaccinated Antibiotic cohort (N=9,531)
All types of antibiotics	4,426 (100.0%)	9,531 (100.0%)
Penicillins	2,636 (59.6%)	5,826 (61.1%)
Cephalosporins	1,478 (33.4%)	1,464 (15.4%)
Tetracycline	316 (7.1%)	49 (0.5%)
Macrolides	1,416 (32.0%)	817 (8.6%)
Aminoglycosides	252 (5.7%)	753 (7.9%)
Polypeptides	2 (0.0%)	17 (0.2%)
Quinolones	148 (3.3%)	92 (1.0%)
Sulfonamides	1,004 (22.7%)	536 (5.6%)
Anti-urinary infectives	257 (5.8%)	157 (1.6%)
Others	132 (3.0%)	162 (1.7%)
Antitubercular agents	28 (0.6%)	40 (0.4%)
Combined regimens	17 (0.4%)	29 (0.3%)
Antitrichomonal/ anticandidal agents	558 (12.6%)	550 (5.8%)
Antifungals	145 (3.3%)	711 (7.5%)

Abbreviations: N, number.
